# Optical Coherence Tomography Angiography Assessed Retinal and Choroidal Microvasculature Features in Patients with Retinitis Pigmentosa: A Meta-Analysis

**DOI:** 10.1155/2019/6723917

**Published:** 2019-11-14

**Authors:** Ling Ling, Feifei Gao, Qinglin Zhang, Tao He, Yi Zhao, Yiqiao Xing, Yifeng Yu, Kaibao Ji

**Affiliations:** ^1^Affiliated Eye Hospital of Nanchang University, Nanchang, Jiangxi, China; ^2^Department of Ophthalmology, Renmin Hospital of Wuhan University, Wuhan, Hubei, China; ^3^Department of Ophthalmology, The Second Affiliated Hospital of Nanchang University, Nanchang, Jiangxi, China

## Abstract

**Background:**

The aim of this study was to assess the retinal and choroidal microvasculature features using optical coherence tomography angiography (OCTA) in patients with retinitis pigmentosa (RP).

**Methods:**

This study was a meta-analysis of relevant published studies that were included after a comprehensive search of PubMed, Embase, Cochrane Library, and Web of Science databases. Mean difference (MD) with a 95% confidence interval was used to assess continuous variable outcomes. Heterogeneity was evaluated using the chi-squared test based on the values of *P* and *I*^2^.

**Results:**

Seven studies were included in this meta-analysis. The vessel density values measured in the superficial and deep foveal zones of RP patients using OCTA were significantly lower than the recorded values in the control groups (MD = −3.58, *P*=0.04; MD = −4.93, *P*=0.02, respectively). The superficial and deep parafoveal vessel density values measured with OCTA were also significantly lower in RP patients than in control groups (MD = −9.09, *P* < 0.00001; MD = −10.74, *P* < 0.00001, respectively); for choriocapillaris vessel density, there was no statistically significant difference between RP patients and controls (MD = −1.33, *P*=0.09). The deep foveal avascular zone (FAZ) was significantly larger in RP patients than in controls (MD = 0.15, *P*=0.01), whereas there was no significant difference in the superficial foveal avascular zones in the two groups (MD = 0.08, *P*=0.11).

**Conclusions:**

We showed that retinal and choroidal vessels were attenuated in RP patients. Additionally, we revealed that the FAZ was larger in RP patients, especially the deep FAZ. OCTA may become a useful modality in the diagnosis and monitoring of patients with RP.

## 1. Introduction

Retinitis pigmentosa (RP) is a kind of inherited retinal disorder characterised by progressive deterioration of the rod and cone photoreceptor cells and the retinal pigment epithelium, which eventually leads to severe impairment of vision [1, 2]. Globally, RP is estimated to occur in 1 in 4000 individuals [3]. The typical signs on the fundus of the RP patient include pale optic nerve head, attenuated retinal vessels, and peripheral bone spicule pigmentation [4]. The most common pathogenesis of RP is mainly associated with different gene mutations [5]; other causes include oxidative stress, Vitamin A deficiency, and immune and inflammatory response [6–8]. Furthermore, reduced retinal blood flow has been reported in RP patients, suggesting altered retinal blood flow to be an underlying factor in the pathology of RP [9, 10].

Optical coherence tomography angiography (OCTA) is a new, noninvasive imaging technique that facilitates the visualisation and quantification of retinal and choroidal circulation without the need for dye injection, offering new insights into the pathogenesis of many retinal and choroidal disorders [11]. Several studies conducted with OCTA have reported reduction of retinal and choroidal blood flow and an increase in the size of the foveal avascular zone (FAZ) in the eyes of RP patients [12, 13]. However, some other studies reported results contrary to these findings [14, 15]. Numerous studies have reported that decreased retinal blood flow was significantly correlated with visual function [16, 17]. To resolve these inconsistencies and ensure clarity on this subject, a comprehensive meta-analysis of published studies is necessary.

To date, there has been no meta-analysis evaluating the retinal and choroidal microvasculature changes measured with OCTA in patients with RP. We thus conducted a much needed meta-analysis to fill this gap and provide ophthalmologists with robust clinical evidence to aid proper management of RP cases.

## 2. Materials and Methods

### 2.1. Literature Search Strategy

**Figure 1:**
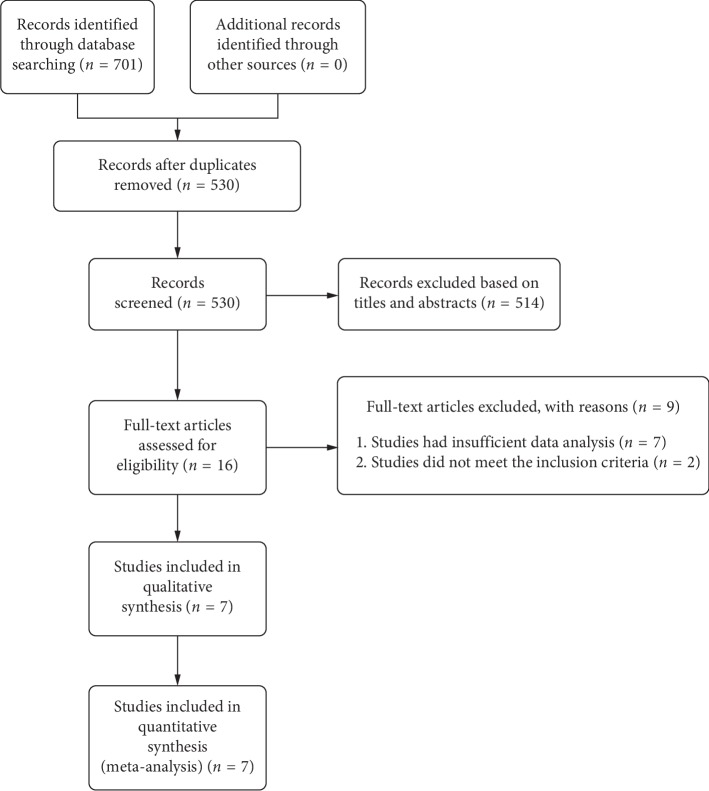
Flow diagram of the article search process for meta-analysis.

We conducted this meta-analysis using previously published studies; no patients were involved in this study; therefore, no informed patient's consent and/or public ethical approval were required. This study was conducted in accordance with the Preferred Reporting Items for Systematic Reviews and Meta-analysis (PRISMA) guidelines [18]. Two independent reviewers (Ling Ling and Kaibao Ji) searched PubMed, Embase, Cochrane Library, and Web of Science databases for all relevant studies published from inception to June 2019. To maximise the number of studies considered, the following search terms were used: “pigmentary retinopathy,” “pigmentary retinopathies,” “retinopathies pigmentary,” “retinitis pigmentosa,” “OCTA,” “optical coherence tomography angiography,” “OCT angiography,” and “optical coherence tomographic angiography.” All articles in English were considered eligible. A final decision was made after the two independent reviewers reached a consensus. The article search steps are illustrated in Figure 1.

### 2.2. Inclusion and Exclusion Criteria

The studies included in the present meta-analysis met the following criteria: (1) original studies; (2) studies provided data on retinal and/or choroidal vascular features; (3) OCTA data were reported as mean ± standard deviation (SD); (4) sample size of the study was at least 10; and (5) primary outcomes in the studies included superficial and deep foveal vessel densities, superficial and deep parafoveal vessel densities, whole choriocapillaris vessel density, and superficial and deep foveal avascular zones.

Studies were excluded based on the following criteria: (1) case reports, abstracts from conferences, posters, animal studies, reviews, and meta-analyses; (2) study objective measures did not meet the inclusion criteria; (3) duplication of the same study; and (4) studies with insufficient data.

### 2.3. Data Extraction and Quality Assessment

Two independent researchers (Ling Ling and Kaibao Ji) independently retrieved and extracted the data from the included studies, and discrepancies were resolved through discussion. The extracted data included the first author, the location of the study, publication year, study design, total number of cases, mean ages, number of males and females, type of OCTA, and outcomes. The methodological quality of case-control studies was assessed with the Newcastle-Ottawa Scale using a score range of 0 to 9 points, with a higher score (NOS ≥ 5) indicating higher study quality [19].

### 2.4. Statistical Analysis

Statistical analysis was performed using Review Manager software version 5.30 (Cochrane Collaboration, Oxford, UK). The continuous variables were summarised as mean ± standard deviation (SD) and mean difference (MD) with a 95% confidence interval (CI) for all effect sizes. The sample mean and standard deviation were calculated as previously described [20, 21]. Heterogeneity was evaluated using the chi-squared test based on the values of *P* and *I*^2^. *I*^2^ values of 25%, 50%, and 75% represented low, moderate, and high heterogeneity, respectively. We selected the random effects model, which is more conservative than the fixed effects model, to collate the data and account for variability both within studies and between studies. A *P* value <0.05 was considered statistically significant.

## 3. Results

### 3.1. Search Results

A total of 701 potentially relevant records published from inception to June 2019 were identified in our literature search (PubMed: 320; Cochrane Library: 8; Embase: 248; Web of Science: 125), of which 171 were duplicates and were therefore excluded from the analysis. After reading the titles and abstracts, 514 more records were excluded. Further reading of the full text of the remaining 16 studies led to the exclusion of 7 studies, which had insufficient data and an additional 2 that did not meet the inclusion criteria. Thus, 7 studies [12–15, 22–24] were eventually included in our meta-analysis (Figure 1).

The seven studies included four cross-sectional studies and three case-control studies; the detailed characteristics and quality assessment of the studies are described in Table 1. A total of 500 eyes (309 patients in RP groups and 191 in control groups) were considered in this meta-analysis.

### 3.2. Main Analysis

#### 3.2.1. Vessel Density Analysis in RP Patients and Controls

**Figure 2:**
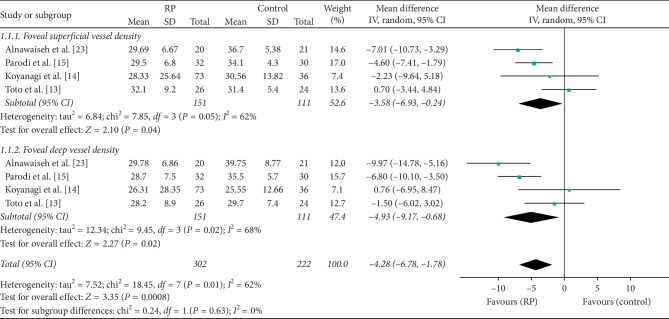
Forest plot of foveal vessel density in RP groups and control groups.

Out of the seven studies evaluated in this meta-analysis, four studies that included 524 eyes (302 eyes in RP and 222 eyes in control) reported on the superficial and deep foveal vessel densities of these eyes. We calculated the mean difference (MD) in superficial foveal vessel density between the RP and control groups, which was −3.58 (95% CI: −6.93 to −0.24, *P*=0.04, Figure 2), indicating that superficial foveal vessel density was lower in RP patients. The MD in deep foveal vessel density between the two groups was −4.93 (95% CI: −9.17 to −0.68, *P*=0.02, Figure 2), revealing that deep foveal vessel density was also reduced in RP patients. The difference between the two groups was statistically significant (MD = −4.28, *P*=0.0008, Figure 2), but there was substantial heterogeneity among the studies for this outcome (chi^2^ = 18.45, *P*=0.01, *I*^2^ = 62%, Figure 2).

**Figure 3:**
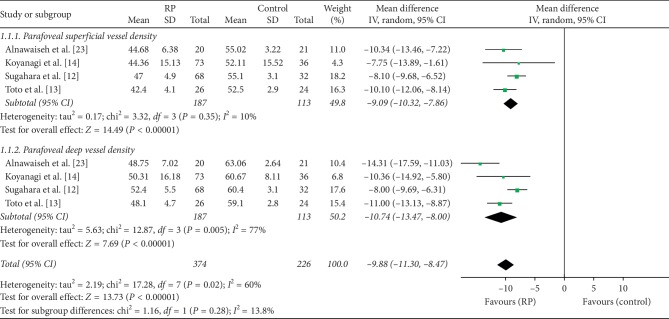
Forest plot of parafoveal vessel density in RP patients and controls.

In addition, four other studies included 600 eyes (374 eyes in RP and 226 eyes in control) and reported on the superficial and deep parafoveal vessel densities of their participants. The MD in superficial parafoveal vessel density between the RP and control groups was −9.09 (95% CI: −10.32 to −7.86, *P* < 0.00001, Figure 3), indicating that superficial parafoveal vessel density was lower in RP patients; no heterogeneity was found among the studies for this outcome (chi^2^ = 3.32, *P*=0.35, *I*^2^ = 10%, Figure 3). The MD in deep parafoveal vessel density between the two groups was −10.74 (95% CI: −13.47 to −8.00, *P* < 0.00001, Figure 3), demonstrating that the deep vessel density was also reduced in RP patients but with substantial heterogeneity among the studies for this outcome (chi^2^ = 12.87, *P*=0.005, *I*^2^ = 77%, Figure 3). The difference between the two groups was significant (MD = −9.88, *P* < 0.00001, Figure 3), but there was substantial heterogeneity found among the studies (chi^2^ = 17.28, *P*=0.02, *I*^2^ = 60%, Figure 3).

**Figure 4:**
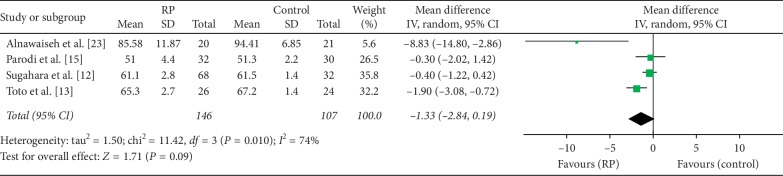
Forest plot for choriocapillaris vessel density in RP patients and controls.

Three of the studies reported on choriocapillaris vessel density and included 253 eyes (146 eyes in RP and 107 eyes in control). The MD in choriocapillaris vessel density between the two groups was −1.33 (95% CI: −2.84 to 0.19, *P*=0.09, Figure 4), indicating that choriocapillaris vessel density was lower in RP patients but not significantly so; there was substantial heterogeneity among the studies for this outcome (chi^2^ = 11.42, *P*=0.01, *I*^2^ = 74%, Figure 4). Subgroup analyses showed that there was no heterogeneity in the macular scan size of 3 × 3 (chi^2^ = 0.01, *P*=0.92, *I*^2^ = 0%, Supplementary [Supplementary-material supplementary-material-1]), but not in macular scan size of 6 × 6 (chi^2^ = 4.98, *P*=0.03, *I*^2^ = 80%, Supplementary [Supplementary-material supplementary-material-1]).

#### 3.2.2. Analysis of the FAZ and Foveal Thickness in RP Patients and Controls

**Figure 5:**
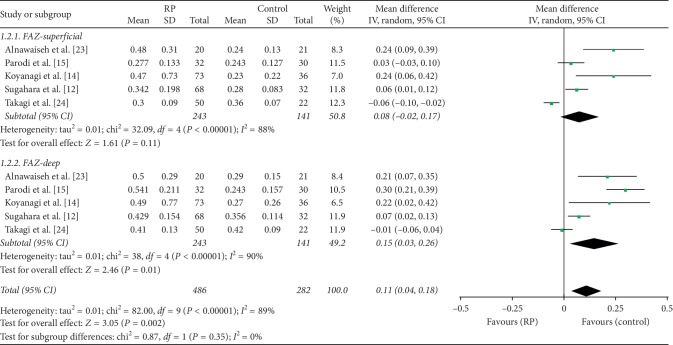
Forest plot for analysis of foveal avascular zone superficial and deep in RP patients and controls.

A total of 486 eyes with RP and 282 control eyes were included in the analysis of the FAZ. The superficial FAZ in RP patients was larger than that of control groups but not significantly so; there was an MD of 0.08 (95% CI: −0.02 to 0.17, *P*=0.11, Figure 5) between the groups, and substantial heterogeneity was found among the studies for this outcome (chi^2^ = 32.09, *P* < 0.00001, *I*^2^ = 88%, Figure 5). However, the deep FAZ in RP patients was significantly larger than that of the controls, with an MD of 0.15 (95% CI: 0.03 to 0.26, *P*=0.01, Figure 5) between the two groups and with substantial heterogeneity among the studies as well (chi^2^ = 38.17, *P* < 0.00001, *I*^2^ = 90%, Figure 5). Subgroup analyses for superficial FAZ found that there was substantial heterogeneity with a macular scan size of 3 × 3 (chi^2^ = 40.81, *P* < 0.00001, *I*^2^ = 93%, Supplementary [Supplementary-material supplementary-material-1]), and there was only one study consisting of a macular scan size of 6 × 6 (Supplementary [Supplementary-material supplementary-material-1]).

**Figure 6:**
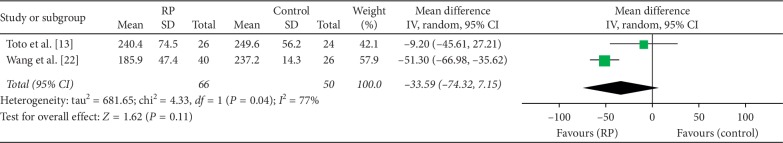
Forest plot for foveal thickness in RP patients and controls.

Foveal macular thickness was lower in RP patients than in controls, with an MD of −33.59 (95% CI: −74.32 to 7.15, *P*=0.11, Figure 6) between the groups and substantial heterogeneity among the studies (chi^2^ = 4.33, *P*=0.04, *I*^2^ = 77%, Figure 6).

## 4. Discussion

To the best of our knowledge, we are the first to conduct a meta-analysis that compares the retinal and choroidal vascular changes measured with OCTA in RP patients and controls. Recently, more studies have reported not only a reduction of retinal and choroidal flow in RP patients, but an expanded FAZ as well [12, 13]. In this study, we pooled the mean foveal and parafoveal vessel densities of study participants, as well as their choriocapillaris vessel densities, FAZ-s, and foveal thicknesses. We revealed that there is significantly reduced vessel density at both the superficial and deep foveal layers in RP patients compared with that of controls. However, we also found the heterogeneity among the included articles to be substantial. Two studies contributed the most to the heterogeneity of this meta-analysis, Toto et al. [13] and Koyanagi et al. [14]. The first reported the highest mean vessel density in RP patients and the highest MD of all the four studies that evaluated foveal superficial vessel density. This could explain the heterogeneity found among the studies, and perhaps, because the authors assessed both eyes of the RP patients, intraobserver variation and bias can occur. The second article (Koyanagi et al.) reported the lowest mean vessel density in RP patients and the highest MD of all the four studies that assessed deep foveal vessel density; this may have contributed the heterogeneity among the studies as well. Moreover, other potentially confounding factors such as age, race, baseline state, and the types of OCTA are unavoidable. We also concluded that parafoveal vessel density is markedly lower in RP patients compared with controls, and the heterogeneity among the studies was significant for the deep vessel density outcome. We observed that the mean vessel density and MD were the highest in the study of Sugahara et al. [12] and that this may have influenced the heterogeneity of the studies. Presently, our findings regarding the blood flow density of retinal vessels are in line with the results presented in the previous literature [23].

Although the photoreceptors and the retinal pigment epithelium (RPE) are recognised as the main sites of pathology in retinal dystrophies, changes in choroidal structure may also play a role in the pathogenesis of this group of diseases; loss of choriocapillaris was detected in vivo human eyes with RP using histopathological analysis [10]. Several studies have also demonstrated that reduced choroidal blood flow occurs in RP [25, 26]. Our results also showed that the choriocapillaris vessel density was lower in the eyes of RP patients than in those of the controls, but the difference was not significant. This may be attributed to the study of Alnawaiseh et al. [23], which had the smallest sample size, and reported the highest mean vessel density both in the RP patients and the controls and had the lowest MD of all the studies. Another possible explanation for this is the variation of methods of quantitative analysis used in these studies. Different macular scan sizes may also contribute the heterogeneity.

We have demonstrated that both the superficial and deep FAZ are larger in RP patients than in controls, especially in the deep FAZ. Previous studies also reported similar results [15, 27]. We considered that two main reasons contributed to the significant heterogeneity observed among the studies included in the present meta-analysis. The first one was that the MD was lowest in the study of Takagi et al. [24]. Another reason may be the significantly reduced vessel density at the deep foveal layer. The following factors may also contribute to the heterogeneity of FAZ: the investigator manually outlined the FAZ, different algorithms for quantitative analysis were used, and different ethnicities and types of studies were included.

The reduction of retinal blood vessels may consequently lead to the thinning of the fovea. We observed that the foveal thickness of RP patients was smaller than that of the controls. Also, we speculated that the small samples may contribute to this significant heterogeneity.

In general, the variations in the different device types may have caused the overall heterogeneity in our meta-analysis. Furthermore, different artefacts can lead to different measurements. Three main artefacts can be distinguished: projection, segmentation, and motion artefacts [28]. Projection artefacts arise from light that is not directly reflected by the moving blood but passes through and illuminates features posterior to the vessel [29]. Projection artefacts are common and affect the diagnostic accuracy of imaging equipment in detecting diseases [30]. Nevertheless, Optovue, Topcon, and Zeiss provide in-built image processing for the removal of projection artefacts [29]. Different segmentation artefacts may also lead to incorrect OCTA results. However, software and hardware improvements are continually evolving to mitigate these limitations [31]. In addition, Enders, et al. demonstrated that 91% of OCTA assessments exhibit acceptable quality for clinical interpretation [28]. Motion artefacts arise from eye movements as very thin white horizontal lines resulting in illusive interruption or displacement of the vessels [28]. Motion artefacts occurred more frequently in the superficial retinal layer. For motion artefacts of the SCP, the Topcon module was superior compared with the other two devices; however, no significant difference among the devices in terms of motion artefacts were detected [32]. In order to minimize motion artefacts, Zeiss employed the Fast-Track and Topcon employed the SMARTTRACK eye tracking system, while Optovue utilized a software-based method in which a retinal area is repeatedly scanned horizontally and vertically [32].

There were several limitations in our meta-analysis. Firstly, the sample size of our study was relatively small and the quality of the included trials was relatively low. Secondly, there was insufficient data on the background information (genotype or genetic characterization) of the RP patients. This is particularly important because different gene mutations may lead to a variety of pathologic phenotypes, including vascular defects. Thirdly, OCTA has several drawbacks including reduced quality of images due to low acuity caused by poor fixation and motion artefacts. Finally, Mastropasqua, et al. [33] have recently reported a reduction in the density of the radial peripapillary capillary network vessels in eyes affected by RP. So, comprehensively quantifying changes in the microvascular density and morphology should be advocated in assessing the pathogenesis of RP. To verify the validity of our meta-analysis, future prospective longitudinal studies of retinal vessels in RP patients need to be conducted.

In summary, our meta-analysis showed that both retinal and choroidal vessels were attenuated in RP patients when compared with controls. Furthermore, we revealed that the FAZ was larger and foveal thickness was smaller in RP patients compared with controls. Finally, our findings suggested that these microvascular parameters may have significant value in the diagnosis and monitoring of disease progression in retinitis pigmentosa.

## Figures and Tables

**Table 1:** Clinical characteristics of the included studies.

Study	Place	Mean age (years)	Study design	Number of eyes (number of patients)	Gender (female/male)	OCTA device	Macular scan size	Foveal and parafoveal VD: inner; outer diameter (mm)	Outcomes of the VD assessed by OCTA	Quality score
Sugahara et al. [12]	Japan	49.9 ± 17.6 54.4 ± 19.9	Cross-sectional study	Cases: 68 (68) Controls: 32 (32)	36/32 20/12	Optovue	3	1; 1–2.5	FAZ-S, FAZ-D, PSVD, PDVD	*∗∗∗∗∗∗*

Toto et al. [13]	Italy	40.1 ± 7.3 42.2 ± 6.5	Cross-sectional study	Cases: 26 (14) Controls: 24 (24)	6/8 10/14	Optovue	6	1; 1–2.5	FT, FSVD, FDVD, PSVD, PDVD, WCVD	*∗∗∗∗∗∗*

Koyanagi et al. [14]	Japan	42.1 ± 25.7 41.2 ± 42.5	Cross-sectional study	Cases: 73 (73) Controls: 36 (36)	36/37 20/16	Optovue	3	1; 1–2.5	FAZ-S, FAZ-D, FSVD, FDVD, PSVD, PDVD	*∗∗∗∗∗∗*

Parodi et al. [15]	Italy	53 ± 18 53 ± 17	Cross-sectional study	Cases: 32 (16) Controls: 30 (30)	6/10 16/14	Topcon	3	None	FAZ-S, FAZ-D, FSVD, FDVD, WCVD	*∗∗∗∗∗∗*

Wang et al. [22]	China	38.7 ± 10.5 42.3 ± 15.7	Prospective case-control study	Cases: 40 (20) Controls: 26 (13)	9/11 8/5	Zeiss	6	1; 1–2.5	FT, FAZ	*∗∗∗∗∗∗*

Alnawaiseh et al. [23]	Germany	42.40 ± 14.11 41.47 ± 13.54	Prospective case-control study	Cases: 20 (20) Controls: 21 (21)	11/9 12/9	Optovue	6	1; 1–2.5	FAZ-S, FAZ-D, FSVD, FDVD, PSVD, PDVD, WCVD	*∗∗∗∗∗∗*

Takagi et al. [24]	Japan	46.8 ± 12.6 50.3 ± 10.0	Case-control study	Cases: 50 (32) Controls: 22 (12)	18/14 11/3	Optovue	3	None	FAZ-S, FAZ-D	*∗∗∗∗∗∗*

FT = Foveal thickness, FAZ-S = Foveal avascular zone superficial, FAZ-D = Foveal avascular zone deep, FSVD = Foveal superficial vessel density, FDVD = Foveal deep vessel density, PSVD = Parafoveal superficial vessel density, PDVD = Parafoveal deep vessel density, WCVD = Whole choriocapillaris vessel density.

## Supplementary Materials

Supplementary Materials Supplementary Figure 1: subgroup analyses for choriocapillary vessel density in different macular scan sizes. Supplementary Figure 2: subgroup analyses for superficial FAZ in different macular scan sizes.
