# Supplementary figures and images for: Optical Coherence Tomography Angiography Assessed Retinal and Choroidal Microvasculature Features in Patients with Retinitis Pigmentosa: A Meta-Analysis
Source: Biomed Res Int. 2019 Nov 14;2019:6723917. doi: 10.1155/2019/6723917 (PMC6881583; doi:10.1155/2019/6723917)

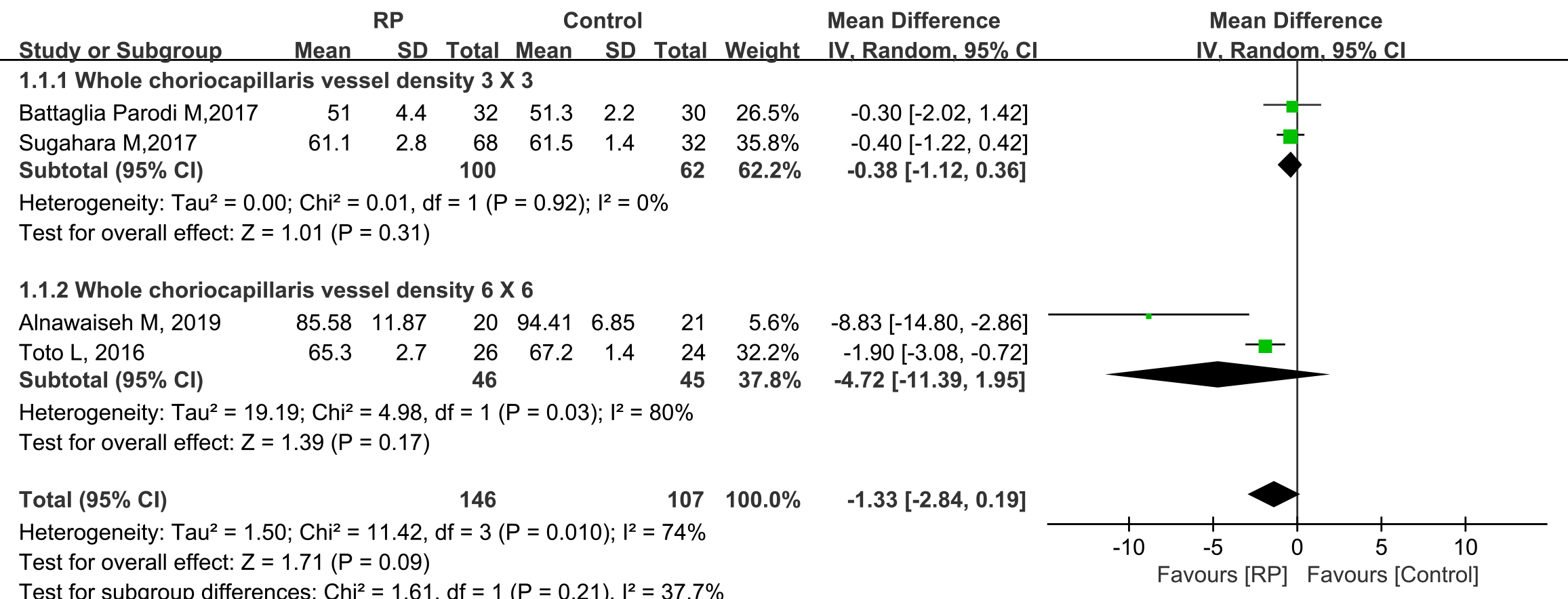

Supplement: Supplementary Materials — Supplementary Figure 1: subgroup analyses for choriocapillary vessel density in different macular scan sizes. Supplementary Figure 2: subgroup analyses for superficial FAZ in different macular scan sizes. [file 6723917.f1.zip › 6723917.f1/Supplementary Figure1 (1).tif]

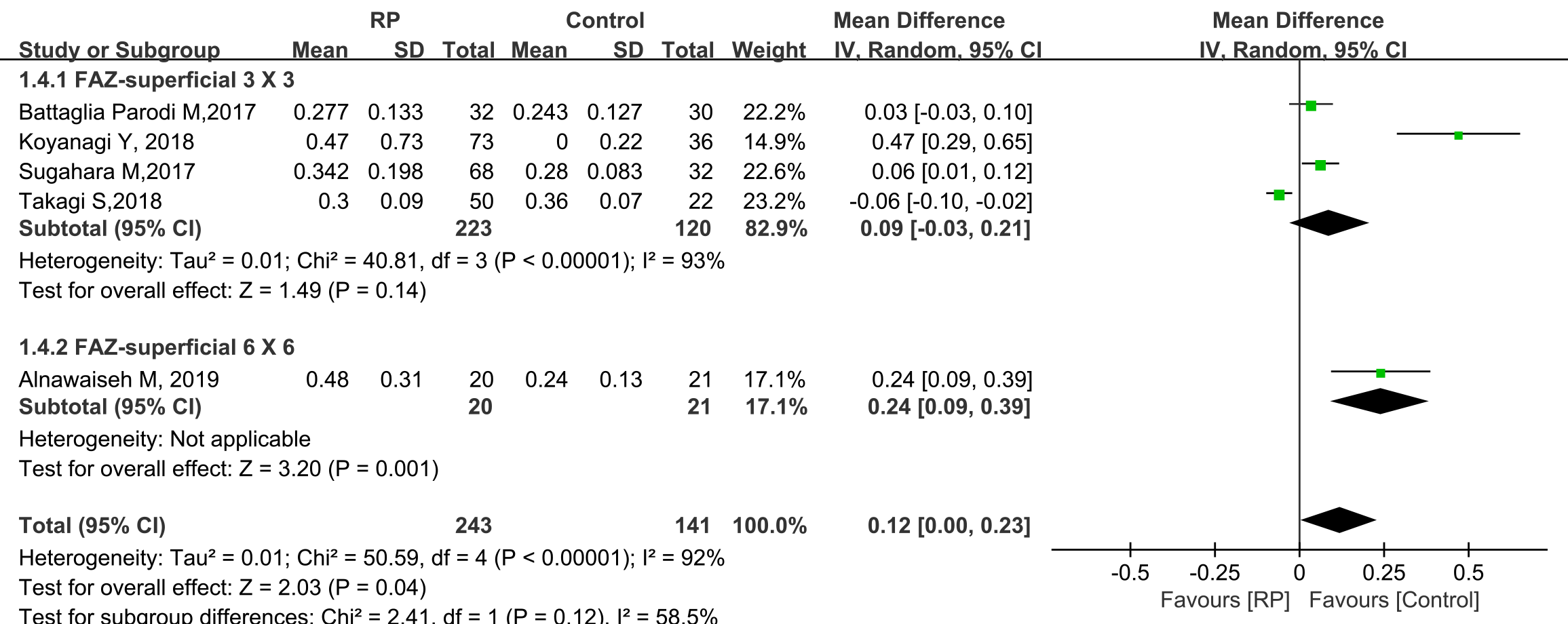

Supplement: Supplementary Materials — Supplementary Figure 1: subgroup analyses for choriocapillary vessel density in different macular scan sizes. Supplementary Figure 2: subgroup analyses for superficial FAZ in different macular scan sizes. [file 6723917.f1.zip › 6723917.f1/Supplementary Figure2.tif]
